# Risk factors and prognostic impact of continuous renal replacement therapy after heart transplantation: a single-center retrospective study

**DOI:** 10.3389/fmed.2026.1807526

**Published:** 2026-04-21

**Authors:** Xiang Wu, Ping-an Lian, Jin-rui Liu, Xi-ran Sun, Qiang Zhou, Lin Li, Chang-an Wang, Jing-hua Zhang

**Affiliations:** 1 Kidney Transplantation and Nephrology Center, Seventh People's Hospital of Zhengzhou, Zhengzhou, Henan, China; 2 Institute of Biological Therapy, Henan Academy of Innovations in Medical Science, Zhengzhou, Henan, China; 3 Department of Cardiology, Seventh People's Hospital of Zhengzhou, Zhengzhou, Henan, China; 4 Department of Cardiovascular Surgery, Seventh People's Hospital of Zhengzhou, Zhengzhou, Henan, China

**Keywords:** acute kidney injury, continuous renal replacement therapy, heart transplantation, perioperative risk factors, prediction model, prognosis

## Abstract

**Background:**

Heart transplantation (HT) is an effective treatment for end-stage heart disease, but postoperative acute kidney injury (AKI) requiring continuous renal replacement therapy (CRRT) is associated with poor outcomes. Although risk factors for AKI after HT have been well established, studies specifically focusing on CRRT as a clinical endpoint and employing rigorous predictive modeling remain limited. This study aims to identify perioperative risk factors for CRRT after HT and to develop a validated prediction model.

**Methods:**

This single-center retrospective study included HT recipients from April 2018 to November 2023. Patients requiring CRRT within 7 days after surgery were compared with those who did not. Candidate predictors were pre-selected based on clinical rationale and previous literature. LASSO regression was used for variable selection to prevent overfitting. A multivariable logistic regression model was then constructed and internally validated using 1,000 bootstrap resamples. Model performance was assessed by discrimination (optimism-corrected AUC), calibration (calibration plot, Hosmer-Lemeshow test), overall fit (Brier score), and clinical utility (decision curve analysis). A time-dependent Cox proportional hazards model was used to evaluate the association between CRRT and postoperative mortality, thereby avoiding immortal time bias.

**Results:**

Among 213 recipients, 30 (14.1%) received CRRT. LASSO regression identified nine key predictors: preoperative hemoglobin, preoperative total bilirubin, preoperative ECMO use, cardiopulmonary bypass time, intraoperative blood loss, red blood cell transfusion volume, mechanical ventilation time, VIS score, and lactate peak. Considering the limited number of events (EPV = 10), three core variables were ultimately included in the multivariable model: preoperative hemoglobin (OR 0.963, 95%CI 0.937–0.986, *p* = 0.003), VIS score (OR 1.282, 95%CI 1.175–1.423, *p* < 0.001), and lactate peak (OR 2.032, 95%CI 1.464–2.986, p < 0.001). The model demonstrated good discrimination (optimism-corrected AUC = 0.885) and excellent calibration (Hosmer-Lemeshow test *p* = 0.678). Decision curve analysis confirmed a positive net benefit across clinically relevant threshold probabilities. After adjusting for confounders, CRRT remained independently associated with increased mortality (adjusted HR = 6.957, 95%CI 3.669–13.192, *p* < 0.001).

**Conclusion:**

Patients requiring CRRT after HT have a markedly poorer prognosis. The internally validated multifactorial model, incorporating preoperative hemoglobin, VIS score, and lactate peak, provides robust predictive value for CRRT and may facilitate early risk stratification. The significant association between CRRT and mortality underscores the critical need for targeted perioperative interventions in high-risk patients.

## 1 Introduction

Heart failure (HF) is a clinical syndrome that seriously threatens human life and health, with its incidence and mortality continuing to rise worldwide. It is estimated that more than 64 million people globally suffer from heart failure, with approximately 8.9 million patients in China (1, 2). Although pharmacological therapy, interventional treatment, and mechanical circulatory support have advanced significantly in recent years, the long-term prognosis of patients with end-stage heart failure remains unsatisfactory. For patients who meet the indications, heart transplantation (HT) is still the most effective treatment for improving survival and quality of life (3). According to the OPTN/SRTR 2021 annual report, the global volume of heart transplantation increased by 67.4% between 2010 and 2021 (4). Meanwhile, post-transplant survival outcomes have improved markedly. Data from the ISHLT registry show that the 1-year and 10-year survival rates after heart transplantation exceed 85 and 50%, respectively (5), and recent studies have further demonstrated that the median postoperative survival has exceeded 12 years (6).

However, with the prolongation of survival among heart transplant recipients, post-transplant–related complications have gradually become important factors affecting patient prognosis. Common complications after heart transplantation include rejection, infection, low cardiac output syndrome, arrhythmias, acute and chronic renal dysfunction, and cardiac allograft vasculopathy (7). Among these, acute kidney injury (AKI) is one of the most common and severe complications after heart transplantation, and its occurrence is closely associated with a significant increase in postoperative mortality. Previous studies have shown that the incidence of AKI after heart transplantation ranges from 14 to 83%, and severe AKI is an independent risk factor for mortality in heart transplant recipients (8, 9). A recent single-center retrospective study also demonstrated that severe AKI after heart transplantation increases the risk of 90-day postoperative mortality in HT recipients (10). Therefore, improving early identification and intervention for AKI after heart transplantation is of great clinical importance for improving patient outcomes.

Renal replacement therapy (RRT) is an important treatment modality for patients with severe AKI (11). Among RRT modalities, continuous renal replacement therapy (CRRT), owing to its superior hemodynamic stability and precise fluid management, has become the most commonly used treatment for critically ill patients, especially those who develop severe AKI after heart transplantation (12, 13). Although CRRT has clear advantages in correcting volume overload and improving metabolic disturbances, its initiation often indicates critical illness and is closely associated with poor prognosis (14).

A recent systematic review and meta-analysis by Khawar et al. (15) comprehensively summarized the established perioperative risk factors for AKI after heart transplantation, identifying age, body mass index, prolonged cardiopulmonary bypass time, ECMO use and extended mechanical ventilation as significant predictors. However, several important gaps remain. First, existing studies have primarily focused on AKI as a composite outcome, whereas investigations specifically dedicated to CRRT as a distinct clinical endpoint are limited. Second, most prior analyses have relied on traditional univariable screening for predictor selection, a method prone to overfitting and potentially omitting important confounders (16). Third, contemporary cohort data on CRRT after heart transplantation in Asian populations remain scarce. Furthermore, a rigorously validated early prediction model specifically for CRRT, incorporating comprehensive perioperative variables and employing modern methodological approaches such as penalized regression and internal validation, has not yet been established.

Therefore, the present study had two primary objectives: (1) to systematically identify perioperative risk factors associated with CRRT initiation after heart transplantation using a methodologically robust approach (LASSO regression) to mitigate overfitting, and (2) to rigorously evaluate the independent prognostic impact of CRRT on patient survival using time-dependent Cox regression analysis. By constructing and internally validating a predictive model, we aim to provide a clinically useful tool for perioperative risk stratification and to inform strategies for improving patient outcomes in this high-risk population.

## 2 Materials and methods

### 2.1 Study design and study population

This study was a single-center retrospective analysis. Clinical data were retrospectively collected from recipients who underwent heart transplantation at Zhengzhou Seventh People’s Hospital between April 2018 and November 2023. All donor hearts were obtained through the China Organ Transplant Response System and complied with relevant national laws and regulations on organ donation. The study protocol was approved by the Medical Ethics Committee of Zhengzhou Seventh People’s Hospital (Approval No. 20231109001). Owing to the retrospective nature of the study, informed consent was waived for all heart transplant recipients.

### 2.2 Inclusion and exclusion criteria

**Figure 1:**
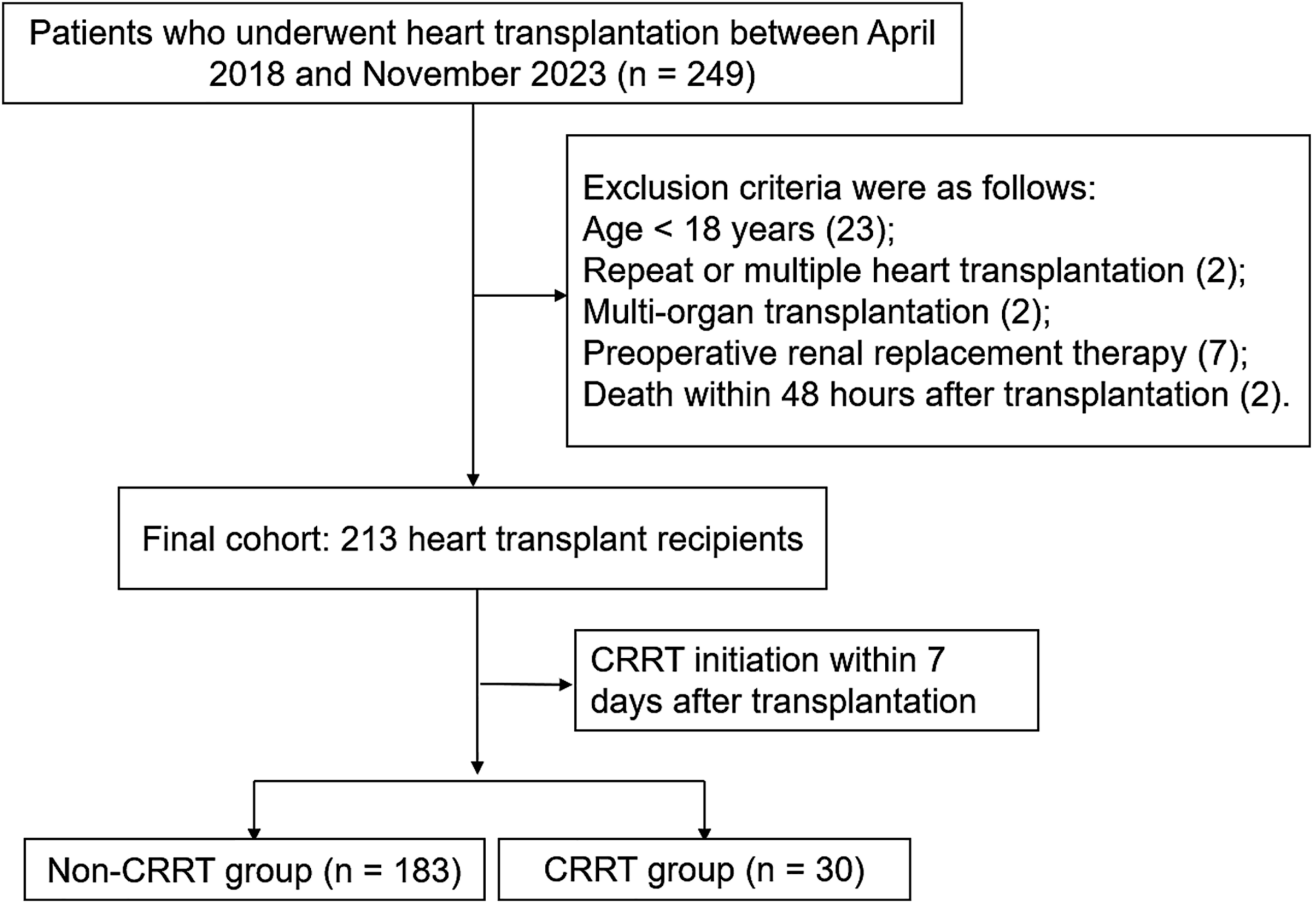
Study flow diagram of patient inclusion and exclusion.

Inclusion criteria: Patients who underwent heart transplantation at Zhengzhou Seventh People’s Hospital between April 2018 and November 2023. Exclusion criteria: Age < 18 years; recipients of second or multiple heart transplantations; patients who underwent concomitant multi-organ transplantation; patients who had received renal replacement therapy within 7 days before surgery; patients who died within 48 h after surgery; and patients with incomplete clinical data for any reason (see Figure 1).

### 2.3 Clinical data collection

#### 2.3.1 Data sources and definitions

Clinical data were obtained from the information system of Zhengzhou Seventh People’s Hospital. Demographic characteristics, medical and surgical histories, laboratory test results, and surgical records were extracted from the Hospital Information System and Electronic Medical Record system. Preoperative left ventricular ejection fraction (LVEF) was obtained from the last preoperative echocardiographic examination. Intraoperative data (including operative records, cardiopulmonary bypass records, anesthesia records, and nursing records) were sourced from the surgical anesthesia system and nursing information system. Preoperative data were defined as the last laboratory results before surgery, and postoperative data were defined as the first laboratory results obtained within 24 h after surgery. Estimated glomerular filtration rate (eGFR) was calculated using the CKD-EPI equation (17).

#### 2.3.2 Preoperative data

Preoperative demographic and clinical data included age, sex, height, weight, and body mass index (BMI). Data on comorbidities and surgery-related history included diabetes mellitus, hypertension, chronic kidney disease (CKD), primary cardiac disease type (coronary artery disease, dilated cardiomyopathy, restrictive cardiomyopathy, hypertrophic cardiomyopathy, and others), history of previous cardiac surgery, pulmonary hypertension, arrhythmias, and the use of intra-aortic balloon pump (IABP). Details of mechanical circulatory support (MCS) bridging strategy, including type (durable LVAD vs. temporary MCS/ECMO) and duration, were also recorded. Durable LVAD duration was defined as the time interval from LVAD implantation to heart transplantation. Device types included EVAHEART I, CH-VAD and other durable LVAD systems. Temporary MCS/ECMO duration was defined as the time from device initiation to transplantation or explantation. For patients transferred from other centers after LVAD implantation, the implantation date was based on external medical records.

Preoperative laboratory parameters included hemoglobin (Hb), red blood cell count (RBC), white blood cell count (WBC), platelet count (Plt), serum creatinine (SCr), blood urea nitrogen (BUN), eGFR, total bilirubin (TBil), aspartate aminotransferase (AST), alanine aminotransferase (ALT), serum albumin (Alb), N-terminal pro–B-type natriuretic peptide (NT-proBNP), and LVEF.

#### 2.3.3 Intraoperative data

Intraoperative data included operative time, cardiopulmonary bypass (CPB) time, aortic cross-clamp (ACC) time, donor heart cold ischemia time, intraoperative blood loss, blood transfusion volume, fluid infusion volume, urine output, autologous blood reinfusion volume, and the dose of methylprednisolone sodium succinate administered intraoperatively. Intraoperative blood loss was estimated by the anesthesia team based on suction canisters and weighed gauze. Urine output was recorded as total volume (mL) and also calculated as mL/kg/h for analysis.

#### 2.3.4 Postoperative data and follow-up

Postoperative data included duration of mechanical ventilation, length of ICU stay, initiation of CRRT within 7 days after surgery, and postoperative BNP and LVEF. For patients who received CRRT, the exact time from ICU admission to CRRT initiation was recorded. To avoid data leakage, all postoperative variables used in the prediction model (e.g., mechanical ventilation time, vasopressor-inotrope score) were defined as values documented before CRRT initiation for cases, and as the first available postoperative values for controls. All patients were followed up, and postoperative survival outcomes were recorded. Patients discharged against medical advice were carefully reviewed; those discharged due to terminal condition or treatment withdrawal were considered as deaths, while others were censored at the time of discharge.

Additionally, key physiological and therapeutic variables were collected to characterize baseline severity. The vasoactive-inotropic score (VIS) was calculated according to the method described by Gaies et al. (18). The maximum VIS within the first 24 h postoperatively was recorded for all patients. For hemodynamic severity markers, lactate peak and mean arterial pressure (MAP) nadir within the first 24 h postoperatively were recorded for all patients. For patients who initiated CRRT within the first 24 h postoperatively, VIS, lactate peak, and MAP nadir were calculated based on data documented prior to CRRT initiation. Regarding nephrotoxic medication exposure, exposure to calcineurin inhibitors, aminoglycosides, vancomycin, or iodinated contrast within 48 h post-surgery was documented. For patients who initiated CRRT within 48 h postoperatively, exposure was recorded up to the time of CRRT initiation. The immunosuppressive regimen after heart transplantation in our center consisted of tacrolimus (target trough concentration 8–12 ng/mL) combined with mycophenolate mofetil and corticosteroids. Calcineurin inhibitors were typically initiated within 24–48 h postoperatively, with dose adjustment based on renal function monitoring.

### 2.4 Diagnosis and staging of AKI, and indications for CRRT initiation

The diagnosis and staging of AKI were based on the 2012 Kidney Disease: Improving Global Outcomes (KDIGO) Clinical Practice Guidelines (19), using a combination of changes in serum creatinine (SCr) and urine output. Baseline SCr was defined as the most recent value prior to surgery, or the admission value if unavailable. Urine output data were obtained from ICU nursing records. CRRT was initiated based on a combination of KDIGO guidelines and clinical judgment by a multidisciplinary team (intensivist, nephrologist, and cardiac surgeon). The primary indications for CRRT initiation in this cohort were systematically reviewed and categorized as: (1) refractory volume overload unresponsive to high-dose loop diuretics (e.g., furosemide equivalent dose > 200 mg/day); (2) severe metabolic acidosis (pH < 7.2) or electrolyte disturbances (e.g., K^+^ > 6.0 mmol/L); (3) progressive oliguria (urine output < 0.3 mL/kg/h for ≥ 24 h) or anuria for ≥ 12 h. The primary CRRT modality was continuous venovenous hemodiafiltration (CVVHDF, multiFiltrate system, Fresenius Medical Care), with regional citrate anticoagulation used in most cases. The prescribed effluent dose was 25–30 mL/kg/h, and the delivered dose was recorded daily. Net ultrafiltration was titrated to achieve a daily negative fluid balance of 500–1,500 mL based on hemodynamic tolerance. The blood flow rate (Qb) was typically set at 150–200 mL/min, titrated according to hemodynamic tolerance.

### 2.5 Statistical analysis

Continuous variables conforming to a normal distribution were expressed as mean ± standard deviation, and comparisons between groups were performed using the independent samples *t*-test. Continuous variables not conforming to a normal distribution were expressed as median (interquartile range), and comparisons between groups were performed using the Mann–Whitney *U* test. Categorical variables were expressed as counts and percentages, and comparisons between groups were performed using the chi-square test or Fisher’s exact test, as appropriate. For missing data, all variables had a missing rate of <5%, so complete-case analysis was used. To assess selection bias, baseline characteristics of included and excluded patients were compared (Supplementary Table S1).

To establish a robust predictive model for CRRT and minimize overfitting given the limited number of events, a two-stage approach was adopted. First, based on clinical theory and findings from previous literature (15), rather than univariate *p*-values, 12 candidate predictors were pre-specified, including: age, preoperative eGFR, preoperative hemoglobin, preoperative total bilirubin, preoperative ECMO use, cardiopulmonary bypass (CPB) time, intraoperative blood loss, intraoperative urine output (mL/kg/h), red blood cell transfusion volume, postoperative mechanical ventilation duration, peak vasoactive-inotropic score (VIS) within 24 h postoperatively, and peak lactate level within 24 h postoperatively. Second, the least absolute shrinkage and selection operator (LASSO) regression was applied to select predictors from the candidate set, using 10-fold cross-validation to choose the optimal penalty parameter (*λ*), and variables with non-zero coefficients were retained (20). The variables selected by LASSO were incorporated into a multivariable logistic regression model to calculate odds ratios (OR) and their 95% confidence intervals (CI), and the variance inflation factor (VIF) was used to assess multicollinearity (VIF > 5 indicated the presence of collinearity). Model performance was evaluated in the following aspects: (1) Discrimination: The area under the receiver operating characteristic curve (AUC) was calculated, and internal validation was performed using 1,000 bootstrap resamples to obtain the optimism-corrected AUC; (2) Calibration: A calibration plot was drawn, and the Hosmer-Lemeshow goodness-of-fit test was used (*p* > 0.05 indicated good calibration); (3) Overall performance: The Brier score was calculated (ranging from 0 to 1, with smaller values indicating better performance); (4) Clinical utility: Decision curve analysis (DCA) was used to assess the net benefit of the model at different threshold probabilities. Survival analysis was performed using the Cox proportional hazards model to evaluate the association between CRRT and postoperative mortality. To avoid immortal time bias, CRRT was treated as a time-dependent covariate, and adjustments were made for age, preoperative eGFR, and CPB time. Results were presented as adjusted hazard ratios (HR) and their 95% CI.

Statistical analyses were performed using R software version 4.2.2 (R Foundation for Statistical Computing). The “glmnet” package was used for LASSO regression, the “rms” package for model calibration, and the “stdca. R” function for decision curve analysis. A two-sided *p* < 0.05 was considered statistically significant.

## 3 Results

### 3.1 Study population and baseline characteristics

**Figure 2:**
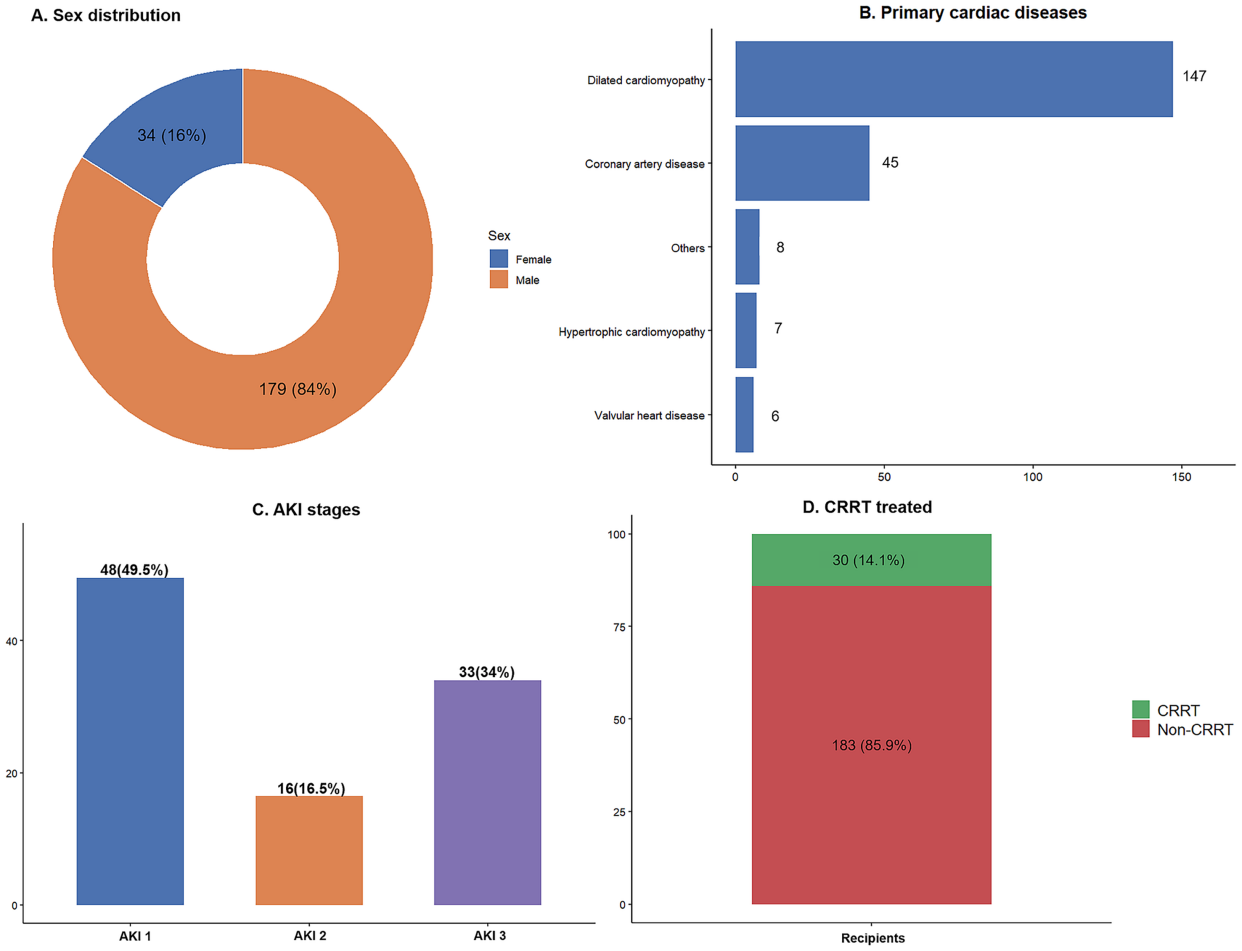
Baseline characteristics and postoperative renal outcomes of heart transplant recipients. **(A)** Sex distribution; **(B)** Composition of cardiac disease etiologies; **(C)** Proportions of AKI stages; **(D)** CRRT group classification.

This retrospective cohort study initially included 249 heart transplant recipients. After applying the inclusion and exclusion criteria, a total of 213 recipients were ultimately included in the analysis. Comparison of baseline characteristics between included patients (*n* = 213) and excluded patients (*n* = 36) showed expected differences in age, sex, and primary disease, primarily due to the exclusion of pediatric patients (age < 18 years, *n* = 18) and those undergoing combined multi-organ transplantation (*n* = 3). The exclusion criteria were applied based on methodological considerations rather than outcomes of interest, suggesting that selection bias is unlikely to materially affect the main findings (Supplementary Table S1). Among the included patients, 179 (84.0%) were male (Figure 2A), with a median age of 50 years (range: 18–75 years). Dilated cardiomyopathy was the most common primary cardiac disease, occurring in 147 patients (69.0%), followed by coronary atherosclerotic heart disease in 45 patients (21.1%). Other etiologies included hypertrophic cardiomyopathy, valvular heart disease, and other types of heart disease (Figure 2B). Among all included patients, the overall incidence of postoperative AKI was 45.5% (97/213). Of these, 49.5, 16.5, and 34.0% of patients were classified as having AKI stage 1, stage 2, and stage 3, respectively (Figure 2C). Patients were divided into the CRRT group (*n* = 30, 14.1%) and the non-CRRT group (*n* = 183, 85.9%) based on whether CRRT was initiated within 7 days postoperatively (Figure 2D).

### 3.2 CRRT practice patterns

Among the 30 patients who received CRRT, the median time from ICU admission to CRRT initiation was 48.0 h (IQR: 26.5–84.0 h), ranging from 12.5 to 168.0 h. The distribution of initiation timing is detailed in Table 1. The primary indications for CRRT initiation were refractory volume overload (16 patients, 53.3%), severe metabolic acidosis/electrolyte disturbances (8 patients, 26.7%), and progressive oliguria/anuria (6 patients, 20.0%). All patients received continuous venovenous hemodiafiltration (CVVHDF) with regional citrate anticoagulation (27 patients, 90%) or heparin anticoagulation (3 patients, 10%). The median prescribed effluent dose was 27.5 mL/kg/h (IQR: 25.0–30.0), and the median delivered dose was 25.1 mL/kg/h (IQR: 23.0–28.5). The median duration of treatment was 5 days (IQR: 3–9 days) (Table 1).

**Table 1:** CRRT practice patterns in heart transplant recipients (*n* = 30).

Parameter	Value
Timing	
Median (IQR), hours	48.0 (26.5–84.0)
Range, hours	12.5–168.0
Initiation within 24 h, *n* (%)	6 (20.0)
Initiation 24–48 h, *n* (%)	9 (30.0)
Initiation 48-72 h, *n* (%)	6 (20.0)
Initiation >72 h, *n* (%)	9 (30.0)
Indications	
Refractory volume overload	16 (53.3)
Severe metabolic acidosis/electrolyte disturbances	8 (26.7)
Progressive oliguria/anuria	6 (20.0)
Modality and anticoagulation	
CVVHDF, *n* (%)	30 (100)
Regional citrate	27 (90)
Heparin	3 (10)
Dose and delivery	
Prescribed, median (IQR)	27.5 (25.0–30.0)
Delivered, median (IQR)	25.1 (23.0–28.5)
Blood flow rate (Qb), mL/min	150–200
Duration	
Treatment duration, days, median (IQR)	5 (3–9)

Data are presented as median (IQR) or *n* (%). Italicized values represent placeholder data. CVVHDF, continuous venovenous hemodiafiltration; IQR, interquartile range.

### 3.3 Univariate analysis of CRRT after heart transplantation

Univariate analysis showed no statistically significant differences between the two groups in terms of sex, age, BMI, or type of primary heart disease (all *p* > 0.05). Regarding preoperative medical history, the CRRT group had a significantly higher prevalence of chronic kidney disease (16.7% vs. 5.5%, *p* = 0.026) and a significantly higher rate of preoperative ECMO use (16.7% vs. 4.4%, *p* = 0.009) compared to the non-CRRT group. Regarding pre-transplant mechanical circulatory support, the CRRT group had a numerically higher rate of any MCS use than the non-CRRT group (26.7% vs. 18.6%), but the difference was not statistically significant (*p* = 0.312). The distribution of MCS types and duration were similar between the two groups (all *p* > 0.05). Regarding preoperative laboratory parameters, the CRRT group had significantly lower hemoglobin levels (115.2 vs. 135.6 g/L, *p* < 0.001) and significantly higher total bilirubin (26.3 vs. 17.7 μmol/L, *p* = 0.002) and NT-proBNP levels (4,205 vs. 2,356 pg/mL, *p* = 0.005). Regarding intraoperative parameters, the CRRT group had significantly longer cardiopulmonary bypass time (179 vs. 128 min, *p* < 0.001) and significantly greater intraoperative blood loss (875 vs. 500 mL, *p* = 0.003), while intraoperative urine output was significantly lower (353 vs. 500 mL, *p* < 0.001). Regarding early postoperative hemodynamic parameters, the CRRT group had a significantly higher peak vasoactive-inotropic score (23.79 ± 5.83 vs. 15.91 ± 5.79, *p* < 0.001) and significantly higher peak lactate level (5.21 ± 1.43 vs. 3.84 ± 1.66 mmol/L, *p* < 0.001), while the lowest mean arterial pressure was significantly lower (53.87 ± 6.09 vs. 75.69 ± 7.73 mmHg, *p* < 0.001). Regarding nephrotoxic drug exposure, the CRRT group had a significantly higher rate of exposure to any nephrotoxic drug (40.0% vs. 23.0%, *p* = 0.048), and the rate of iodinated contrast exposure was also significantly higher (20.0% vs. 8.2%, *p* = 0.043). Regarding postoperative recovery parameters, the CRRT group had significantly longer mechanical ventilation duration (52.9 vs. 18.0 h, *p* < 0.001) and significantly longer ICU length of stay (211.5 vs. 119.5 h, *p* < 0.001) compared to the non-CRRT group Table 2.

**Table 2:** Comparison of baseline characteristics and intraoperative/postoperative parameters between heart transplant recipients with and without CRRT.

Parameter	Non-CRRT (*n* = 183)	CRRT (*n* = 30)	*p* value
Sex [male, *n* (%)]	156 (85.2)	23 (76.7)	0.234
Age (years)	50.0 (38.0,58.0)	49.0 (40.8,58.3)	0.877
Height (cm)	170.2 ± 6.7	170.9 ± 8.1	0.835
Weight (kg)	65.0 (57.0,74.0)	62.0 (56.5,76.5)	0.995
BMI (kg/m^2^)	22.6 (20.6,26.1)	22.5 (21.1,26.0)	0.886
Primary disease [*n* (%)]			
Coronary artery disease	36 (19.7)	9 (30.0)	0.199
Valvular heart disease	5 (2.7)	1 (3.3)	0.854
Cardiomyopathy	137 (74.9)	18 (60.0)	0.090
Other heart diseases	5 (2.7)	3 (10.0)	0.263
Preoperative history			
Hypertension	32 (17.5)	3 (10.0)	0.305
Diabetes mellitus	36 (19.7)	8 (26.7)	0.380
Chronic kidney disease	10 (5.5)	5 (16.7)	0.026
Arrhythmia	106 (57.9)	16 (53.3)	0.638
History of IABP use	77 (42.1)	14 (46.7)	0.638
History of ECMO use	8 (4.4)	5 (16.7)	0.009
Pre-transplant MCS bridging [*n* (%)]			
Any MCS	34 (18.6)	8 (26.7)	0.312
Durable LVAD	13 (7.1)	2 (6.7)	1.000
Temporary MCS/ECMO	21 (11.5)	6 (20.0)	0.228
MCS duration (days), median (IQR)			
Durable LVAD	86 (24–189)	82 (19–176)	0.845
Temporary MCS/ECMO	9 (5–15)	10 (6–18)	0.562
Preoperative parameters			
eGFR (mL/min·1.73 m^2^)	105.9 (80.0,131.2)	94.6 (68.1,146.0)	0.138
LVEF (%)	26.0 (21.0,31.0)	27.5 (23.5,35.3)	0.052
NT-proBNP (pg/mL)	2,356 (1,007,4,849)	4,205 (2,260,7,286)	0.005
WBC (10^9^/L)	6.5 (5.6,8.1)	7.3 (6.4,10.3)	0.037
RBC (10^12^/L)	4.5 ± 0.7	3.9 ± 0.7	0.001
Plt (10^9^/L)	190.8 ± 65.6	164.1 ± 86.7	0.051
Hb (g/L)	135.6 ± 19.7	115.2 ± 17.0	<0.001
SCr (μmol/L)	75.0 (63.0,92.0)	81.5 (66.0,95.5)	0.249
BUN (mmol/L)	8.0 (6.4,10.4)	8.7 (6.2,12.8)	0.456
Uric acid (μmol/L)	415.0 (310.0,487.7)	384.6 (288.1,528.5)	0.653
Alb (g/L)	39.0 (36.4,41.3)	38.0 (35.5,41.1)	0.223
TBil (μmol/L)	17.7 (12.7,26.0)	26.3 (15.0,55.4)	0.002
ALT (U/L)	26.0 (16.0,40.0)	20.5 (14.8,35.2)	0.396
AST (U/L)	25.0 (20.0,37.0)	27.0 (19.0,73.3)	0.357
PRA [n (%)]	10 (5.5)	3 (10.0)	0.336
Intraoperative parameters			
Operative time (min)	250.0 (215.0,293.0)	326.5 (281.3,487.5)	<0.001
Aortic cross-clamp time (min)	44.0 (38.0,51.0)	47.0 (41.8,54.3)	0.049
CPB time (min)	128 (113,160)	179 (150,292)	<0.001
Donor cold ischemia time (min)	308.0 (117.0,361.0)	349.0 (238.389.5)	0.069
Blood loss (mL)	500(500,600)	875 (500–1,000)	0.003
Fluid infusion (mL)	1,100 (800–1,500)	1,100 (925–1,300)	0.509
Urine output (mL)	500 (302–860)	388 (172–688)	0.049
Urine output (ml/kg*h)	1.81 (1.06–3.41)	0.97 (0.39–1.79)	<0.001
Intraoperative defibrillation [*n* (%)]	39 (21.3)	9 (30.0)	0.291
Red blood cell transfusion (U)	0 (0,0)	0 (0,4)	<0.001
Platelet transfusion (U)	0 (0,0)	0 (0,0.25)	0.072
Cryoprecipitate transfusion (U)	0 (0,0)	0 (0,1.5)	0.017
Methylprednisolone dose (g)	0.5 (0.5,0.5)	0.5 (0.44,0.5)	0.063
Nephrotoxic exposure within 48 h [*n* (%)]	42 (23.0)	12 (40.0)	0.048
Calcineurin inhibitors	35 (19.1)	3 (10.0)	0.215
Aminoglycosides	8 (4.4)	4 (13.3)	0.058
Vancomycin	12 (6.6)	5 (16.7)	0.067
Iodinated contrast	15 (8.2)	6 (20.0)	0.043
Postoperative parameters			
Mechanical ventilation time (h)	18.0 (13.0,28.0)	52.9 (20.9,141.9)	<0.001
ICU length of stay (h)	119.5 (112.7,146.4)	211.5 (139.2,429.8)	<0.001
Maximum VIS	15.91 ± 5.79	23.79 ± 5.83	<0.001
Peak lactate (mmol/L)	3.84 ± 1.66	5.21 ± 1.43	<0.001
MAP nadir (mmHg)	75.69 ± 7.73	53.87 ± 6.09	<0.001
Postoperative LVEF (%)	62 (57,67)	62 (55,66)	0.400
BNP (pg/mL)	686 (370,1,023)	n (367,1,125)	0.450

Data are presented as *n* (%), mean ± standard deviation, or median (interquartile range). MCS, mechanical circulatory support; LVAD, left ventricular assist device; ECMO, extracorporeal membrane oxygenation; IABP, intra-aortic balloon pump; CPB, cardiopulmonary bypass; VIS, vasoactive-inotropic score; MAP, mean arterial pressure; ICU, intensive care unit. For patients who initiated CRRT within 24 h postoperatively, the peak VIS, peak lactate level, and lowest MAP were calculated based on data recorded before CRRT initiation. Pp values marked with * were calculated using Fisher’s exact test due to small expected frequencies.

### 3.4 Variable selection and multivariable analysis

**Figure 3:**
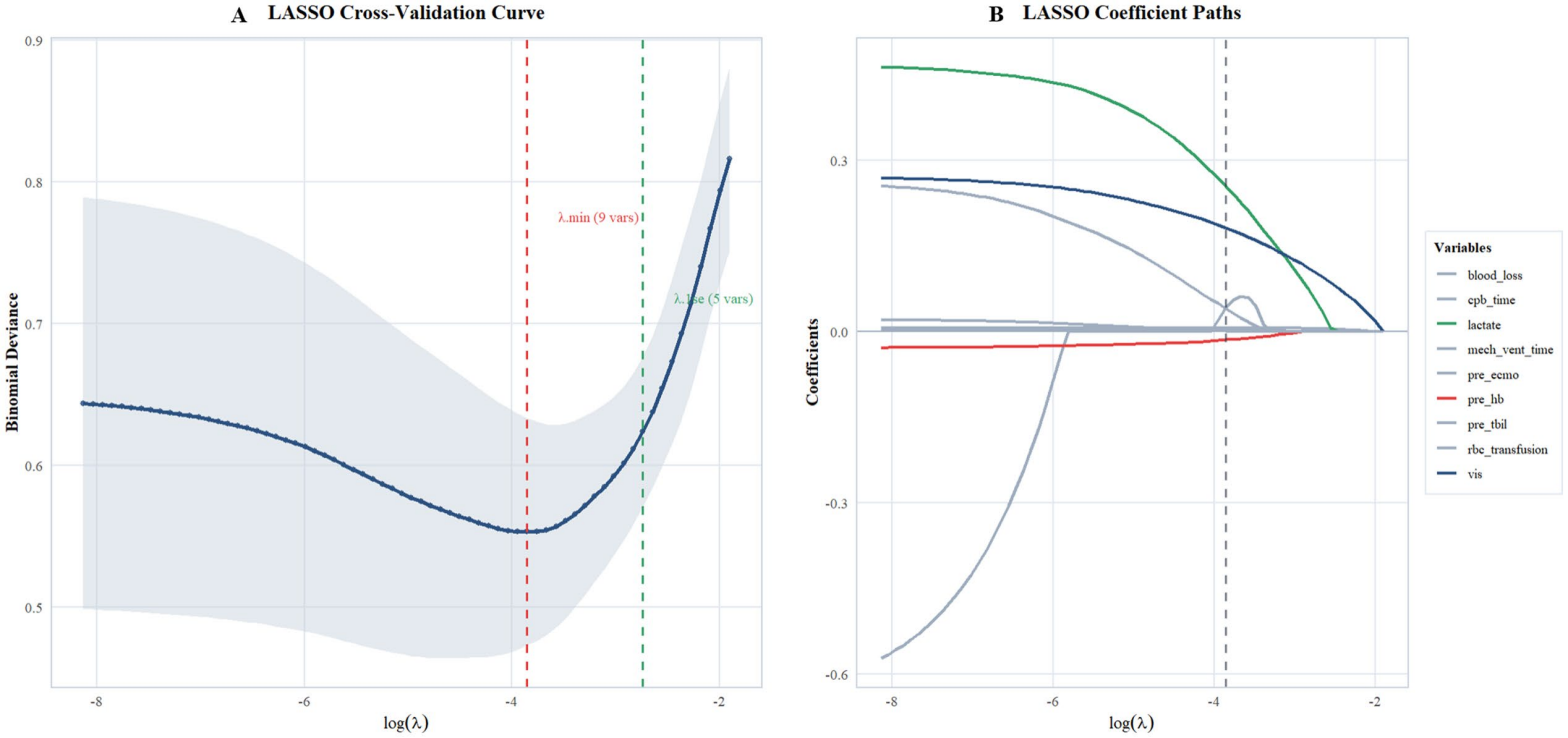
Variable selection using LASSO regression. **(A)** Plot of partial likelihood deviance versus log(*λ*). The left vertical line indicates the optimal λ value (λ.min). **(B)** LASSO coefficient profiles of the 12 candidate predictors. The vertical line is drawn at the selected *λ* min.

From the 12 prespecified candidate predictors, 9 variables with non-zero coefficients at the optimal *λ* value were selected using LASSO regression with 10-fold cross-validation, including: preoperative hemoglobin, preoperative total bilirubin, preoperative ECMO use, cardiopulmonary bypass time, intraoperative blood loss, red blood cell transfusion volume, mechanical ventilation duration, vasoactive-inotropic score, and peak lactate level (Figures 3A,B). Based on the LASSO selection results and statistical power considerations (30 CRRT events, EPV = 10), three core variables were ultimately included in the multivariable logistic regression model: preoperative hemoglobin, vasoactive-inotropic score, and peak lactate level.

Multivariable logistic regression analysis showed that preoperative hemoglobin level (OR 0.963, 95% CI 0.937–0.986, *p* = 0.003), vasoactive-inotropic score (OR 1.282, 95% CI 1.175–1.423, *p* < 0.001), and peak lactate level (OR 2.032, 95% CI 1.464–2.986, *p* < 0.001) were all independent influencing factors for CRRT initiation. Among these, preoperative hemoglobin level was a protective factor, while vasoactive-inotropic score and peak lactate level were risk factors (Table 3). Multicollinearity was assessed using the variance inflation factor (VIF), and the results showed that VIF values for all variables were less than 1.3, indicating no significant multicollinearity among the variables.

**Table 3:** Multivariable logistic regression analysis of CRRT after heart transplantation.

Parameter	*β*	SE	Wald *χ^2^*	*p*	OR	95% CI	VIF
Preoperative hemoglobin	−0.038	0.0129	8.737	0.003	0.963	0.937–0.986	1.043
VIS score	0.2482	0.0483	26.421	<0.001	1.282	1.175–1.423	1.282
Peak lactate level	0.7091	0.1799	15.535	<0.001	2.032	1.464–2.986	1.265

OR indicates the change in CRRT risk for each one-unit increase in the variable. SE, standard error; CI, confidence interval; VIF, variance inflation factor.

### 3.5 Performance and validation of the prediction model

**Figure 4:**
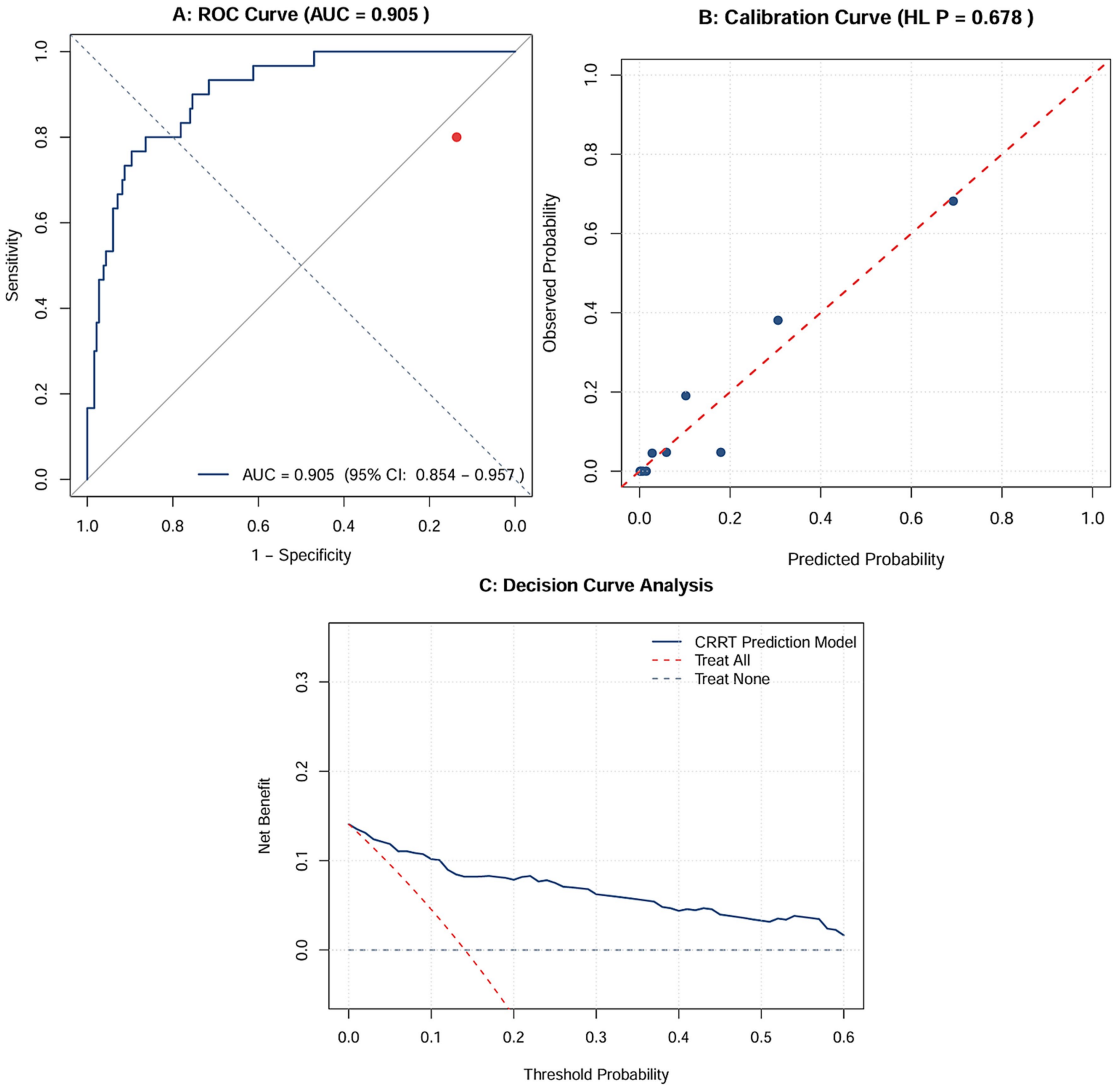
Performance evaluation of the CRRT prediction model. **(A)** ROC curve showing the discriminative ability of the model and the AUC (95% confidence interval); **(B)** Calibration curve comparing the predicted probability of CRRT occurrence with the actual observed probability, along with the Hosmer–Lemeshow test result; **(C)** Decision curve analysis (DCA) showing the net benefit of the model across different threshold probabilities, compared with the “intervene for all” and “intervene for none” strategies.

The final multivariable logistic regression model (including preoperative hemoglobin, VIS score, and peak lactate level) demonstrated good discriminative ability. Receiver operating characteristic (ROC) curve analysis showed that the apparent AUC of the model was 0.905 (95% CI: 0.854–0.957) (Figure 4A). ROC analysis of individual predictors showed that the apparent AUCs for preoperative hemoglobin, VIS score, and peak lactate level were 0.694, 0.833, and 0.734, respectively (Table 3). After internal validation using 1,000 bootstrap resamples, the optimism-corrected AUC was 0.885, with an optimism of 0.02, indicating robust model discrimination. The calibration curve showed good overall agreement between predicted probabilities and observed probabilities (Figure 4B), and the Hosmer-Lemeshow goodness-of-fit test was not statistically significant (*χ^2^* = 5.725, *p* = 0.678). The calibration intercept was −0.0002 (ideal value 0), and the calibration slope was 1.001 (ideal value 1), suggesting good model calibration. The Brier score was 0.076, reflecting a small overall prediction error. Decision curve analysis (DCA) showed that within a threshold probability range of 1 to 60%, the net benefit of using this model to guide clinical decisions was superior to the strategies of “intervening for all” or “intervening for none” (Figure 4C), with a maximum net benefit of 0.141 (at a threshold of 0), indicating that the model has good clinical utility Table 4.

**Table 4:** ROC curve analysis of CRRT after heart transplantation.

Parameter	Apparent AUC	Cutoff value	Sensitivity	Specificity
Preoperative hemoglobin	0.694	118	0.500	0.852
VIS score	0.833	17.4	0.867	0.699
Peak lactate level	0.734	3.75	0.900	0.525
Combined model	0.905	0.197	0.800	0.863

AUC, area under the curve; cutoff values were determined based on the Youden index.

### 3.6 Impact of CRRT on survival outcomes

**Figure 5:**
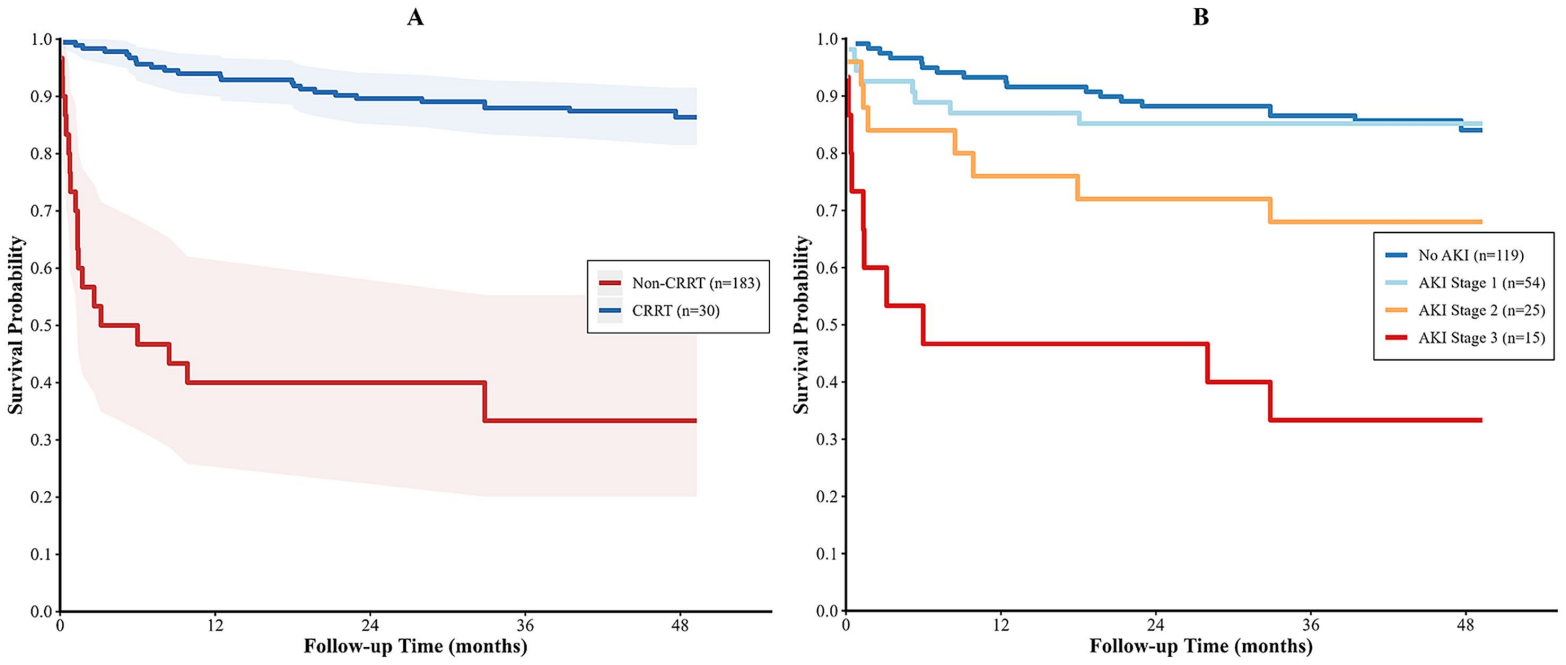
Survival analysis. **(A)** Kaplan–Meier survival curves for the CRRT group and the non-CRRT group, showing a statistically significant difference in cumulative survival rates between the two groups (log-rank *p* < 0.001); **(B)** Kaplan–Meier survival curves stratified by AKI stage, showing a negative correlation between AKI severity and patient survival (log-rank *p* < 0.001).

All 213 heart transplant recipients were included in the survival analysis. By the end of follow-up, the overall survival rate was 78.9% (168/213). The mortality rate in the CRRT group was 66.7% (20/30), which was significantly higher than that in the non-CRRT group (13.7%, 25/183). The median survival time in the CRRT group was 1.3 months (95% CI: 0.8–6.0 months), whereas the median survival time in the non-CRRT group was not reached. Kaplan–Meier survival curves showed that the difference in cumulative survival rates between the two groups was statistically significant (log-rank *p* < 0.001) (Figure 5A). When patients were stratified by AKI stage, survival rates showed a stepwise decline with increasing AKI severity. The 3-year survival rates were 89.1% in the no AKI group, 83.3% in the AKI stage 1 group, 84.0% in the AKI stage 2 group, and 40.0% in the AKI stage 3 group (*p* < 0.001) (Figure 5B). After adjusting for age, preoperative eGFR, and cardiopulmonary bypass time in a multivariable Cox proportional hazards regression model, CRRT remained independently associated with increased postoperative mortality (HR = 6.957, 95% CI: 3.669–13.192, *p* < 0.001) (Table 5).

**Table 5:** Cox regression analysis of the association between CRRT and mortality after heart transplantation.

Variable	HR	95% CI	*p* value
CRRT	6.957	3.669–13.192	<0.001
Age	1.012	0.985–1.040	0.400
Preoperative eGFR	0.992	0.981–1.004	0.190
CPB time	1.005	1.002–1.009	0.0049

HR, hazard ratio; CI, confidence interval; eGFR, estimated glomerular filtration rate; CPB, cardiopulmonary bypass.

## 4 Discussion

In this single-center retrospective cohort study of 213 heart transplant recipients, we found that 14.1% of patients required CRRT within 7 days postoperatively, and these patients had significantly worse survival outcomes. Using LASSO regression for variable selection and bootstrap internal validation, we constructed a parsimonious prediction model incorporating three preoperative and early postoperative variables: preoperative hemoglobin (protective factor), vasoactive-inotropic score (VIS), and peak lactate level (both risk factors). The model demonstrated excellent discrimination (optimism-corrected AUC = 0.885), good calibration (Hosmer-Lemeshow test *p* = 0.678), and showed a positive net benefit across clinically relevant threshold ranges in decision curve analysis. Importantly, after adjusting for confounding factors and treating CRRT as a time-dependent covariate to avoid immortal time bias, initiation of CRRT remained independently associated with an approximately sevenfold increase in postoperative mortality (adjusted HR = 6.957, 95% CI: 3.669–13.192), which is consistent with previous reports linking severe AKI requiring dialysis after heart transplantation to poor outcomes (21).

The mechanisms underlying AKI after heart transplantation are complex and involve multiple factors, including insufficient preoperative renal functional reserve, perioperative hemodynamic instability, ischemia–reperfusion injury, and inflammatory responses (22–24). In this study, the overall incidence of AKI was 45.5%, which is broadly consistent with the range of 40%–70% reported in previous studies, indicating that AKI remains one of the most common and clinically significant complications after heart transplantation (8). Notably, a considerable proportion of patients progressed to stage 3 AKI, reflecting severe renal dysfunction in some recipients during the postoperative period; this may be attributed to the inclusion of patients with more severe underlying conditions in our cohort. AKI is one of the independent predictors of early-to-mid-term mortality in heart transplant recipients. As a key treatment modality for moderate-to-severe AKI, the initiation of CRRT typically indicates critical illness and is associated with poor outcomes (21). Previous studies have reported that the incidence of CRRT after heart transplantation ranges from 5.8 to 33.7% (25–27). The incidence of CRRT in this study was 14.1%, which is generally consistent with previous reports. Furthermore, the survival rate of the CRRT group was significantly lower than that of the non-CRRT group, suggesting that the need for CRRT after heart transplantation is an important marker of poor prognosis.

Our findings extend the existing literature in several respects. First, unlike previous studies that focused on AKI as a composite outcome, we specifically examined CRRT as a clinical endpoint representing severe renal dysfunction requiring therapeutic intervention. Second, we employed rigorous statistical methods (LASSO regression, bootstrap internal validation, and decision curve analysis) to address overfitting and evaluate clinical utility. Third, our cohort provides contemporary data from an Asian population, which has been underrepresented in previous studies on this topic. The three variables retained in the final model reflect distinct pathophysiological pathways contributing to post-transplant renal vulnerability. Multiple meta-analyses have confirmed that preoperative anemia is associated with an increased incidence of AKI after cardiac surgery (28, 29). The emergence of preoperative hemoglobin as a protective factor aligns with the established relationship among anemia, decreased oxygen-carrying capacity, and renal ischemic injury. Anemia is common in patients with end-stage heart failure and may promote the development and progression of AKI by reducing blood oxygen-carrying capacity, thereby exacerbating renal tissue hypoxia and oxidative stress (29–31). During cardiopulmonary bypass, hemodilution can further reduce hemoglobin levels and aggravate hypoxia in the renal medulla (31, 32). Notably, we adjusted for intraoperative blood loss and transfusion volume in the candidate predictor set, suggesting that the predictive value of hemoglobin may extend beyond merely reflecting perioperative blood management and may instead represent baseline physiologic reserve. In this study, when preoperative hemoglobin level was ≤118 g/L, the risk of CRRT increased significantly, indicating that assessment and management of perioperative anemia may have important clinical value.

The VIS quantifies the cumulative exposure to vasopressors and inotropes and serves as an effective surrogate for hemodynamic instability and low cardiac output syndrome, having been shown to accurately predict mortality after cardiac surgery (33). Although vasoactive agents improve cardiac output and contractility, enhancing perfusion to vital organs, they also increase myocardial oxygen consumption and induce blood flow redistribution, leading to inadequate supply to other organs and exacerbating metabolic acidosis, myocardial ischemic necrosis, renal dysfunction, and peripheral circulatory failure. In this context, a higher VIS reflects more severe hemodynamic impairment and can be regarded as a reasonable clinical marker for assessing cardiovascular status following cardiac surgery (34). Similarly, peak lactate level, as a marker of tissue hypoperfusion and metabolic stress, further substantiates that systemic hemodynamic compromise—rather than isolated cardiac or renal dysfunction—drives severe AKI requiring renal replacement therapy. Importantly, by strictly restricting the measurement of predictors to the period prior to CRRT initiation, concerns regarding temporal validity and data leakage have been addressed. For patients who received CRRT within 24 h postoperatively, VIS, lactate, and hemodynamic parameters were all calculated using data recorded before CRRT initiation, ensuring that the model is applicable for early risk stratification. The three variables retained in the final model—preoperative hemoglobin, VIS score, and peak lactate level—reflect distinct, clinically accessible pathophysiological pathways. Preoperative hemoglobin represents baseline physiologic reserve and oxygen-carrying capacity; VIS score quantifies the cumulative burden of hemodynamic compromise; and lactate peak captures the severity of tissue hypoperfusion and metabolic stress. Together, these variables enable bedside risk stratification using routinely available clinical data. Notably, refractory volume overload was the predominant indication for CRRT initiation in our cohort (53.3%), underscoring the importance of venous congestion in the pathogenesis of severe AKI after heart transplantation. Preoperative total bilirubin, which emerged as a predictor in LASSO selection, likely serves as a surrogate marker of chronic hepatic congestion secondary to right heart failure and venous congestion, reflecting the severity and duration of pre-transplant hemodynamic compromise. This interpretation is consistent with the clinical observation that volume overload, rather than metabolic disturbances or oliguria, was the primary trigger for CRRT initiation in most patients.

Our survival analysis confirms the strong association between post-transplant CRRT and increased mortality. However, we emphasize that this relationship likely reflects indication bias—that is, CRRT identifies patients with severe multi-organ dysfunction—rather than demonstrating that CRRT itself causes adverse outcomes. By using time-dependent Cox regression, we minimized immortal time bias and provided a more accurate estimate of the association. The adjusted HR of 6.957 should be interpreted as the magnitude of elevated risk associated with the clinical trajectory requiring CRRT, rather than a causal effect of the treatment itself. The identification of modifiable risk factors through our model suggests several actionable intervention strategies for high-risk patients. For patients with low preoperative hemoglobin, optimizing anemia management before transplantation may enhance renal resistance to injury. Intraoperative strategies, including minimizing blood loss, shortening cardiopulmonary bypass time, and maintaining adequate perfusion pressure, may mitigate renal ischemic injury. Postoperatively, early recognition of hemodynamic compromise through close monitoring of VIS and lactate trends enables timely intervention.

### 4.1 Strengths and limitations

The main strengths of this study include the use of LASSO regression to address overfitting, internal validation via bootstrap resampling, and assessment of calibration and clinical utility through decision curve analysis. Detailed reporting of CRRT practice patterns enhances reproducibility. Nevertheless, several limitations should be acknowledged. The single-center retrospective design limits generalizability, as patient characteristics, CRRT initiation thresholds, and perioperative management may differ across institutions. Although internal validation via bootstrap resampling supports model stability, external validation in independent cohorts has not been performed and is needed before clinical deployment of this model. Additionally, the small number of CRRT events (*n* = 30) necessitates cautious interpretation, and the model should be considered exploratory pending external validation. Finally, detailed echocardiographic assessment of right ventricular function and invasive hemodynamic measurements were not systematically available, so we could not directly quantify the severity of venous congestion; preoperative total bilirubin was used as a surrogate marker.

## 5 Conclusion

In conclusion, heart transplant recipients requiring postoperative CRRT represent a distinct high-risk subgroup with significantly reduced survival. Our internally validated prediction model (which requires external validation before clinical application), incorporating preoperative hemoglobin, VIS score, and peak lactate level, may aid in early identification of patients at high risk for severe AKI requiring renal replacement therapy, but external validation is needed before clinical application. While the association should not be interpreted as causal, these findings underscore the importance of comprehensive perioperative optimization and close monitoring in this vulnerable population.

## Data Availability

The original contributions presented in the study are included in the article/Supplementary material, further inquiries can be directed to the corresponding authors.
