# Supplementary material for: Risk factors and prognostic impact of continuous renal replacement therapy after heart transplantation: a single-center retrospective study
Source: Front Med (Lausanne). 2026 Apr 21;13:1807526. doi: 10.3389/fmed.2026.1807526 (PMC13138926; doi:10.3389/fmed.2026.1807526)
Supplement: Supplementary file 1 [file Table_1.DOCX]

Supplemental Table 1. Comparison of baseline characteristics between included and excluded patients.

| Parameter | Included (n = 213) | Excluded (n = 36) | P value |
| --- | --- | --- | --- |
| Age, years, median (IQR) | 50.0 (38.0, 58.0) | 16.0 (14.0, 44.8) | <0.001 |
| Age < 18 years, n (%) | 0 (0) | 19 (52.8) | <0.001 |
| Sex, male, n (%) | 179 (84.0) | 24 (66.7) | 0.015 |
| Primary disease, n (%) | | | |
| Dilated cardiomyopathy | 147 (69.0) | 12 (33.3) | <0.001 |
| Coronary artery disease | 45 (21.1) | 3 (8.3) | 0.071 |
| Valvular heart disease | 6 (2.8) | 1 (2.8) | 0.999 |
| Other heart diseases | 15 (7.0) | 20 (55.6) | <0.001 |
| Exclusion reason, n (%) | | | |
| Age < 18 years | - | 19 (52.8) | - |
| Preoperative CRRT | - | 10 (27.8) | - |
| Combined multi-organ transplantation | - | 3 (8.3) | - |
| Second or multiple transplantation | - | 1 (2.8) | - |
| Incomplete clinical data | - | 3 (8.3) | - |
| Postoperative death within 48 hours | - | 0 (0) | - |

**Supplementary Material 2: Prediction Model Specification**

**Full multivariable logistic regression equation:**

Logit(P) = **-5.15832**+ (-0.03798) × (Preoperative hemoglobin, g/L) + 0.24820 × (VIS score) + 0.70908 × (Peak lactate, mmol/L)

Where P is the probability of CRRT initiation within 7 days after heart transplantation.

**Example calculation:**

For a patient with: Preoperative hemoglobin = 120 g/L; VIS score = 25; Peak lactate = 4.0 mmol/L.

| Component | Calculation | Value |
| --- | --- | --- |
| Intercept | -5.15832 | -5.15832 |
| Hemoglobin | -0.03798 × 120 | -4.5576 |
| VIS score | 0.24820 × 25 | 6.205 |
| Lactate | 0.70908 × 4.0 | 2.83632 |
| **Logit(P)** | **Sum** | **-0.6746** |

P = 1 / (1 + e^0.6746) = 1 / (1 + 1.963) = 1 / 2.963 = **0.34 (34%)**

**Interpretation:** This patient has an estimated 34% probability of requiring CRRT within 7 days post-transplantation.

**Note on model use:** This prediction model was derived from a single-center retrospective cohort (n=213, CRRT events=30) and has been internally validated using 1000 bootstrap resamples (optimism-corrected AUC = 0.885). External validation in independent cohorts is required before clinical application. The model is intended to assist risk stratification, not to dictate clinical decision-making. The intercept and coefficients are specific to the study population and time period; recalibration may be needed when applied to different settings.
